# Clinical Presentation and Physiotherapy Rehabilitation of Cauda Equina Syndrome with Urinary Incontinence: A Case Report

**DOI:** 10.7759/cureus.70236

**Published:** 2024-09-26

**Authors:** Samruddhi Aherrao, Priya Tikhile, Pratik Phansopkar, Ratnakar Ambade

**Affiliations:** 1 Department of Cardiorespiratory Physiotherapy, Ravi Nair Physiotherapy College, Datta Meghe Institute of Higher Education and Research, Wardha, IND; 2 Department of Musculoskeletal Physiotherapy, Ravi Nair Physiotherapy College, Datta Meghe Institute of Higher Education and Research, Wardha, IND; 3 Department of Orthopedics, Jawaharlal Nehru Medical College, Datta Meghe Institute of Medical Sciences, Wardha, IND

**Keywords:** cauda equina syndrome, low back pain, physiotherapy, range of motion, urinary incontinence

## Abstract

Compression of the lumbar spine's nerve roots results in the uncommon but dangerous illness known as cauda equina syndrome (CES), which impairs motor, sensory, and autonomic functions. To relieve pressure on the cauda equina, immediate surgical decompression is essential. However, long-term rehabilitation is frequently necessary for recovery, with physiotherapy playing a crucial role. Human vertebrae are a powerful, complex anatomical structure. Spinal injuries may be the cause of restrictions in daily activities. In the back and sacral regions, the nerve roots continue as the cauda equina. The lower limbs and pelvic organs communicate with each other through these nerves, which transmit and receive messages. CES is an uncommon but potentially fatal disease that results from the cauda equina being displaced in the spinal canal. The malfunctioning of many lumbar and sacral nerve roots of the cauda equina is the cause of CES. This is a case study of a male 8-year-old who complained of low back pain, bilateral lower limb weakness, and urine incontinence when he visited the hospital. History of the patient revealed that he had a history of falling to the ground two years ago from a height of around four feet. In this case report, the role of physiotherapy is to improve the posture and back muscle strength in cauda equina patients.

## Introduction

Cauda equina syndrome (CES) is a condition with a very high level of medico-legal profile. It usually manifests after a significant central lumbar disc protrusion, sequestration, or herniation [1]. CES is a common but fatal illness, apart from back pain. Radicular indications and symptoms such as lower limb soreness, neurological abnormalities, saddle sensory anomalies, and malfunction of the bladder, intestine, and sexual organs are often associated with a medical disease. Delays in diagnosing and treating CES may lead to post-compression poor mental health, an increased risk of depression, and chronic severe anxiety [2]. Because it has the potential to inflict severe bodily and psychological harm, CES, which is associated with lumbar disc herniation, is a serious instability [3]. Disc changes include spondylosis, disc degeneration, lumbar osteoarthritis, and degenerative disc disease, collectively referred to as spondylosis. Osteophytes change the nerves, and pain can result from degeneration of the vertebrae and lumbar spine. Backaches can be caused by osteophytes, or bone spurs, which can vary in size and frequency. The likelihood of developing this condition increases with age [4]. Additional causes include lumbar vertebrae fractures, spinal tumors, and any spinal treatment that causes these issues. The patient reports experiencing incontinence, gluteal pain, lower limb paralysis, and low back pain. The patient's daily activities are all restricted as a result. More than half of patients have low back discomfort, which lowers their quality of life and makes it difficult for them to adopt any position [5].

Studies show that more than 160000 Americans suffer spinal cord injuries annually. Approximately 64% of spinal injuries happened in the thoracolumbar region. One considers a spinal fracture to be a fatal injury. The most prevalent causes were collisions (45%), injuries (20%), injuries from sports (15%), assault (15%), as well as additional causes (5%) [6]. Lumbar segmental instability is characterized by a loss of spinal flexibility and stiffness, which can result in discomfort, spinal deformity, or neurological dysfunction [7]. Many clinical signs, including saddle hyperesthesia or anesthesia, reduced anal and bulbocavernosus reflexes, malfunctioning of the bladder and rectal sphincter, and sexual impotence, are suggestive of conus or cauda syndrome. Other symptoms frequently associated with CES, such as sciatica, continuous back pain, lower limb motor weakness, and disability of medial plantar and Achilles tendon reflexes, were not considered incomplete because they were not thought to be pathognomonic for this symptom complication [8]. A variety of clinical symptoms may arise from the breakdown of the spinal cord, lumbosacral nerves, and anterior motor units caused by trauma to the thoracolumbar spine. Pain, temperature, loss of touch, reduced vibratory and proprioceptive perceptions, fine and gross motor function, and neurologic dysfunction can all appear in the lower extremities [9]. In this instance, physiotherapy guided body mechanics, posture, and lifestyle modifications to help prevent more issues and improve overall health. CES patient education is critical in providing an understanding of their disease. Thus, the aim of this case report is to improve posture and strengthen back muscles, enhance the quality of life, and reduce urinary incontinence in patients with CES through physiotherapy.

## Case presentation

Patient information

**Figure 1:**
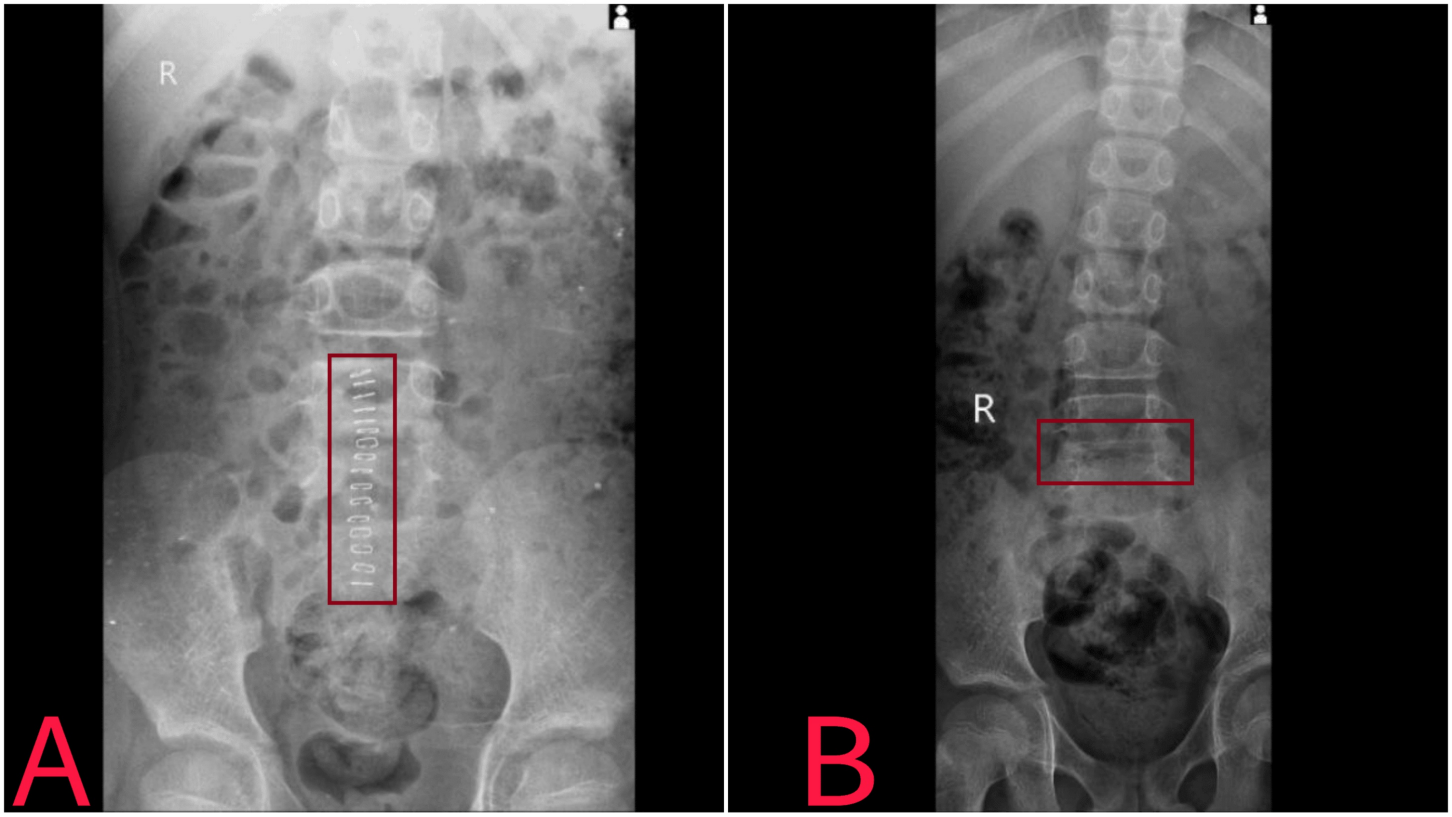
X-ray of the spine Figure A: Post-X-ray shows that posterior decompression and spinal fusion have been performed. Figure B: Pre-X-ray reveals reduced vertebral body height at the L5 and S1 levels, sclerotic changes, degenerative changes in the intervertebral space, and a decrease in two interspinous processes.

An 8-year-old boy complained of both lower limb paralysis and severe back discomfort when he arrived at the Acharya Vinobha Bhave Rural Hospital. According to the patient's medical history, he fell two years ago from four feet of height. After that, he was sent to a nearby hospital, where the doctor gave him some medicine, and no more tests or investigations were carried out. The same day, he was sent back home. He took many weeks off from rest as the pain kept him from moving from his bed. He had trouble executing ordinary tasks like walking and sitting for extended periods. Therefore, As he returned to AVBRH after two months, radiographic examinations revealed a lesion in the spinal canal at the L5 and S1 levels, most likely due to the cauda equina. In addition, he reported having intermittent urine incontinence and tingling in both feet. As a result, the procedure for posterior decompression and spinal fusion was scheduled (Figure 1).

Clinical findings

Before the examination began, the patient and his parents gave their informed consent, and the patient was examined. He was supportive and aligned with the place, person, and time in his immediate vicinity. The patient was observed lying in a supine position with the lower limb supported by a cushion underneath. His vitals are stable, and he is afebrile. The pain was pointing in the surrounding area, and on the numerical pain rating scale, it was 8/10 during movement and 3/10 during relaxation. He has grade 2 tenderness over the lower back region. All the superficial sensations are intact, and deep sensations are impaired. He has weakness in the bilateral lower limb with manual muscle testing grade 2. He has a reduced range of motion (ROM) of the bilateral lower limb. The Modified Oswestry Low Back Pain Disability Questionnaire, the Pelvic Floor Impaired Questionnaire, and the Pediatric Balance Scale (PBS) were utilized to evaluate functional balance skills and pelvic floor dysfunctions (PFDs).

Modified Oswestry Disability Index

The Modified Oswestry Disability Index (MODI) is the most widely used outcome measure for low back pain. Ten components make up a well-validated self-report questionnaire for minor back pain. There is a maximum score of five for each category. The section score is zero if the first statement is marked and five if the final item is marked. Lower scores indicate better functioning. The total score is expressed as a percentage. This study sought to determine whether practicing filling out the MODI forms improved scoring accuracy [10].

Pelvic Floor Impaired Questionnaire

The term PFDs refers to a broad range of linked clinical illnesses, including urogenital symptoms, prolapse of the pelvic organs (POP), bowel incontinence (FI), incontinence of urine (UI), sex issues, and others. It has seven questions, each of which must be answered three times using the scales that were previously mentioned. It considers symptoms in the vagina or pelvis, colon or rectum, bladder or urine, and their effects on function, mental health, and social health over the last three months. The responses range from "not at all" (0) to "quite a bit" (3) for each question. Scale scores are obtained by individually computing the means of the three scales. The questionnaires were used to evaluate urinary incontinence or PFD for the general public [11].

Pediatric Balance Scale

Developed to assess balance in school-aged children with mild to severe motor deficits, the PBS is a modified version of Berg's Balance Scale [12]. The fourteen components on the scale are graded as 0 (lowest function) to 4 (highest function), with a maximum score of 56 points.

Sensory Examination

A clinical evaluation that assesses the nerve system's sensory functioning is called a neurological sensory assessment. A series of tests are used to evaluate the state and functionality of the senses, including touch, pain, vibration, temperature, position awareness, and position perception. The main components of a neurological sensory assessment are outlined below [13]. Table 1 and Table 2 show sensory examination on both the upper and lower limb of both sides.

**Table 1:** Superficial sensory examination of the left and right side

Superficial sensations on the upper limb and lower limb	Left	Right
Pain	Present	Present
Touch	Present	Present
Temperature	Present	Present
Pressure	Present	Present

**Table 2:** Deep sensory examination for left and right side

Deep sensations on the upper limb and lower Limb	Left	Right
Proprioception	Impaired	Present
Kinesthesia	Impaired	Present
Vibration	Impaired	Impaired

Range of motion

The pre-ROM assessment of the lower limb is presented in Table 3.

**Table 3:** Pre-ROM assessment of the lower limb NA: not applicable; ROM: range of motion

Joint/ movement	Right (active)	Right (passive)	Normal ROM	Left (active)	Left (passive)	Endfeel
Hip flexion	0-40	0-50	0-120	0-50	0-70	Empty
Hip extension	NA	NA	0-30	NA	NA	NA
Hip abduction	0-10	0-15	0-25	0-10	0-15	Empty
Hip adduction	0-10	0-15	0-30	0-10	0-15	Empty
Knee flexion	0-50	0-60	0-135	0-30	0-40	Empty
Knee extension	NA	NA	0-135	NA	NA	NA
Ankle dorsiflexion	0-20	0-20	0-20	0-20	0-20	Firm
Ankle plantarflexion	0 30	0-30	0-35	0-30	0-30	Firm

Manual muscle testing 

Manual muscle testing of the right and left side of the lower limb is given in Table 4.

**Table 4:** Manual muscle testing for right and left lower limb ROM: range of motion

Muscles	Right		Left	
Hip flexors	2+/5	Moves through a partial ROM	2+/5	Moves through a partial ROM
Hip extensors	2/5	Moves through a complete ROM	2/5	Moves through a complete ROM
Hip abductors	2+/5	Moves through a partial ROM	2+/5	Moves through a partial ROM
Knee flexors	2+/5	Moves through a partial ROM	2/5	Moves through a complete ROM
Knee extensors	3/5	Holds test position (no added pressure)	3/5	Holds test position (no added pressure)
Ankle dorsiflexors	5/5	Holds test position against strong pressure	5/5	Holds test position against strong pressure
Ankle plantarflexors	5/5	Holds test position against strong pressure	5/5	Holds test position against strong pressure

Physiotherapy intervention

The phasic physical therapy intervention is shown in Table 5, the post-manual muscle testing assessment is shown in Table 6, and the outcome measures used are shown in Table 7. Table 8 shows the post-treatment ROM.

**Table 5:** Intervention with dosage ROM: range of motion

Time	Goal	Intervention	Rationale	Dosage
Day 1-Week	Educate the patient	Teaching the patient about his condition and the intended physical therapy treatment	Allows the patient to take an active role in their healing process and ensures that the treatment plan is followed	
	To relieve pain	The patient received adequate posture advice in both sitting and laying positions, which helped to relieve discomfort and provide relaxation	To help manage pain	Every 2 hours
	To improve the ranges	Functional ROM exercises were shown for the following: hip flexion, extension, abduction, and adduction; knee flexion and extension; ankle dorsiflexion and plantar flexion. Active movements of both upper extremities were taught to maintain joint range	To restore patients' functional ability and mobility	10 reps with 10 sec hold-1 set
	To improve strength	Starting with static quadriceps and static hamstring exercises in a prone position, isometric workouts for the muscles of the quadriceps and hamstrings were taught. In addition to these protocols, core strengthening was used. Initially, the back and stomach muscles were taught solely isometric motions	The stability, balance, and posture that are improved by strengthening the upper limbs are essential for good gait mechanics. It also promotes weight transfer and improves coordination, enhancing gait efficiency	10 reps with 10 sec hold-1 set
	To improve bed mobility	Bedside sitting and log rolling are initiated	To reduce bedsores and contractures	Every 3 hours of a day
	To improve upper limb strengthening	TheraBand and dumbbells are used for upper limb and lower limb strengthening workouts	To improve strength and endurance	10 reps with 10 sec hold-1 set
	To reduce pulmonary complications	Breathing exercises should be initiated with incentive spirometry	It helps reduce pulmonary complications and improve lung function	10 reps with 10 sec hold-1 set
Day 8-3 week	To restore muscle strength in the back	Strength training for the back and abdominals, as well as resistant lower-limb movements and pelvic floor muscle exercises	To improve muscle ability and muscle strength	10reps with 10sec hold -1 set
	To treat urinary incontinence	Interferential current is given as it is frequently used to activate muscles, lessen pain, and accelerate healing. Kegels exercises are advised, and pelvic bridging exercises are given	To improve muscle contraction and reduce pain	15 minutes twice a day. 10 reps with 10 sec hold-1 set
	To improve endurance	Progressive Resistance Exercises are given, such as dumbbell curls, bench presses, squats, and wall pushes. Progress mechanical resistance with Therabands and weight cuffs	It aids in overall recovery and enables individuals to regain their ability to engage in daily activities and return to their desired level of physical function	10reps with 10sec hold -1 set
	To improve and maintain posture	Postural correction exercises are given. Splint-like postural belts and lumbar belts are used	Helps to maintain posture and regain it properly	Twice a day
	To improve stabilization	Core stabilization exercises help to enhance trunk control and stability	To stabilize the trunk	3 times a day

**Table 6:** Post-operative manual muscle testing ROM: range of motion

Muscles	Right		Left	
Hip flexors	4/5	Holds test position against slight pressure	3/5	Holds test position (no added pressure)
Hip extensors	2/5	Moves through a complete ROM	3/5	Holds test position (no added pressure)
Hip abductors	4/5	Holds test position against slight pressure	3/5	Holds test position (no added pressure)
Knee flexors	4/5	Holds test position against slight pressure	3/5	Holds test position (no added pressure)
Knee extensors	3/5	Holds test position (no added pressure)	4/5	Holds test position against slight pressure
Ankle dorsiflexors	5/5	Holds test position against strong pressure	4/5	Holds test position against slight pressure
Ankle plantarflexors	5/5	Holds test position against strong pressure	4/5	Holds test position against slight pressure

**Table 7:** Outcome measures with follow-up PBS: Pediatric Balance Scale

Sr. No	Outcomes	On first day( post-operative)	On week two (post-operative)
1	Visual analog scale	8/10 (severe pain)	3/10 (mild pain)
2	Numerical pain rating scale	8/10 (severe pain)	4/10 (mild pain)
3	Lower extremity functional scale	10/100 (severe function limitation)	52/100 (moderate function limitation)
4	PBS	16/56 (severe balance impairment)	30/56 (moderate balance impairment)
5	Pelvic floor impaired questionnaire	120/200 (severe impairment)	50/200 (mild impairment)
6	Oswestry Disability Index	34/50 (moderate disability)	15/50 (mild disability)

**Table 8:** Post-ROM assessment of lower limb NA: not applicable; ROM: range of motion

Joint/ movement	Right (active)	Right (passive)	Normal ROM	Left (active)	Left (passive)	Endfeel
Hip flexion	0-100	0-110	0-120	0-80	0-90	Empty
Hip extension	NA	NA	0-30	NA	NA	NA
Hip abduction	0-25	0-25	0-25	0-25	0-25	Empty
Hip adduction	0-30	0-30	0-30	0-20	0-25	Empty
Knee flexion	0-120	0-130	0-135	0-120	0-120	Empty
Knee extension	NA	NA	0-135	NA	NA	NA
Ankle dorsiflexion	0-20	0-20	0-20	0-20	0-20	Firm
Ankle plantarflexion	0-35	0-35	0-35	0-35	0-35	Firm

## Discussion

A rare but dangerous disorder known as CES is brought on by compression of the cauda equina, a bundle of nerves near the base of the spinal cord. Reduced mobility, bowel, and bladder problems, and sensory and motor deficiencies are all possible outcomes of CES. Patients with CES benefit greatly from physiotherapy, particularly following surgical decompression or other medical procedures. Physiotherapy plays a vital role in the rehabilitation process of individuals with cauda equina illness, targeting the diverse range of motor, sensory, and functional impairments resulting from the illness. Improved mobility, pain management, and general quality of life are achieved by patients with early intervention, customized exercise regimens, and regular follow-up, all of which lead to better long-term outcomes.

The condition known as CES arises when the lumbosacral nerve roots are compressed by the thecal sac of the lumbar vertebra [14]. The two main muscle groups in the body that maintain the stability of the spine are the core muscles, according to their characteristics and roles. The deep core muscles, also known as the local stabilizing muscles, make up the first set of muscles. The transversus abdominis, lumbar multifidus, internal oblique muscle, and quadratus lumborum comprise the majority of these muscles. The transverse abdominal ligament contracts simultaneously with the lumbar multifidus, which is directly linked to each lower spinal segment. With contractions, abdominal draw-in preserves the neutral zone of the spine and offers segmental stabilization [15]. Lumbar segmental stability is a crucial biomechanical factor that influences the symptoms experienced by individuals with mechanical low back pain [16]. The dorsiflexion muscles of the lower limbs and extremities are referred to as the "posterior chain." These muscles support the lumbar and thoracic spines on the dorsal side of the body, as well as peripheral joints like the ankle, knee, and hip [17]. Two main clinical groups can be distinguished from CES: partial CES, which includes the case study in this article and is characterized by a lack of urge to urinate or difficulty urinating but no urine retention, and CES with a history of documented urine retention [18]. It is common to have both chronic pain and depression concurrently. People who suffer from chronic pain often exhibit signs of depression or other mental health issues. People who are depressed frequently express discomfort. Depression rates among patients with chronic pain have been observed to range from 10% to 100%. The majority of research indicates that percentage rates vary from 30% to 60% [19]. According to Gabriel's research, post-operative physical therapy enhances confidence and gait. For the lower limbs, an energy-based technique is offered that helps reduce discomfort and increase strength, flexibility, and ROM. In cases of traumatic post-operative rehabilitation, physical therapy can help maintain and improve strength and mobility [20]. There are many different ways to treat low back pain; initially, the patient's back discomfort was treated using a pathoanatomical method. These markers aid in recognizing the condition's symptoms and indicators. Early physiotherapy increases the ROM and reduces pain. Thermotherapy produces calmness and pain relief. Common at-home workouts to improve strength and mobility include walking and bridging [21].

Early management is critical for improving functional outcomes and preventing irreparable brain damage in people with CES. Physical therapy is crucial for enhancing recovery and lowering the risk of long-term damage in the early stages of treatment. Physiotherapy is essential for people with CES because it increases the body's mobility and functional abilities. The patient's psychological health is enhanced by ambulation and gait training, which help to restore the patient's confidence and balance. It lessens difficulties down the road. The patient's strength, sensibility, and ROM all improve; these factors may be beneficial when developing a rehabilitation program for those with CES. Physiotherapy can help a patient regain optimal weight distribution, improve endurance, health, joint mobility, and proprioception, eliminate the underlying cause of a physical alteration, improve clinical symptoms to resume normal functioning, reduce the need for NSAIDs, relieve pain, and extend the patient's quality of life. Regaining confidence and balance is possible for patients who get ambulation and gait training, which benefits their mental health. When creating a rehabilitation program for individuals with CES, all of these exercises can be helpful.

## Conclusions

After serious traumas, CES is a typical side effect that requires medical attention from a multidisciplinary team. A patient who has had a good rehabilitation would be able to easily perform daily duties. In this case, an 8-year-old male with a lesion in the spinal canal at L5 and S1 underwent surgery for posterior decompression and spinal fusion. In this case, we conclude that after physiotherapy rehabilitation, he was able to resume daily activities. Thus he had improved his back muscle strength and posture due to rehabilitation.

## References

[1] Gardner A, Gardner E, Morley T (2011). Cauda equina syndrome: a review of the current clinical and medico-legal position. Eur Spine J.

[2] Kimber D, Pigott T (2023). Cauda equina screening in physiotherapy: a qualitative study of physiotherapists in a community musculoskeletal service: are we asking the right questions and are we asking the questions right?. Musculoskelet Sci Pract.

[3] McCarthy MJ, Aylott CE, Grevitt MP, Hegarty J (2007). Cauda equina syndrome: factors affecting long-term functional and sphincteric outcome. Spine.

[4] Shamrock A, Donnally C, Varacallo M (2023). Lumbar spondylolysis and spondylolisthesis. Star pearls.

[5] Joshi A, Chitale N, Phansopkar P (2022). The impact of physical therapy rehabilitation on pain and function in a patient with cauda equina syndrome. Cureus.

[6] Hitwar V, Yadav V, Vaidya L, Jain M, Wadhokar O (2022). Rehabilitation of lumbar spine fracture with pedicel screw fixation and posterior decompression and fusion. J Med P’ceutical Allied Sci.

[7] Davarian S, Maroufi N, Ebrahimi I, Farahmand F, Parnianpour M (2012). Trunk muscles strength and endurance in chronic low back pain patients with and without clinical instability. J Back Musculoskelet Rehabil.

[8] Wostrack M, Shiban E, Obermueller T, Gempt J, Meyer B, Ringel F (2014). Conus medullaris and cauda equina tumors: clinical presentation, prognosis, and outcome after surgical treatment: clinical article. J Neurosurg Spine.

[9] Radcliff KE, Kepler CK, Delasotta LA (2011). Current management review of thoracolumbar cord syndromes. Spine J.

[10] Mehra A, Baker D, Disney S, Pynsent PB (2008). Oswestry Disability Index scoring made easy. Ann R Coll Surg Engl.

[11] Zuchelo LT, Bezerra IM, Da Silva AT (2018). Questionnaires to evaluate pelvic floor dysfunction in the postpartum period: a systematic review. Int J Womens Health.

[12] Franjoine MR, Gunther JS, Taylor MJ (2003). Pediatric balance scale: a modified version of the berg balance scale for the school-age child with mild to moderate motor impairment. Pediatr Phys Ther.

[13] Freeman C, Okun MS (2002). Origins of the sensory examination in neurology. Semin Neurol.

[14] Williams JT, Lister H, Fakouri B, Panchmatia JR (2024). The national suspected cauda equina syndrome pathway: implications for physiotherapists. Physiotherapy.

[15] Chang WD, Lin HY, Lai PT (2015). Core strength training for patients with chronic low back pain. J Phys Ther Sci.

[16] Kumar SP (2011). Efficacy of segmental stabilization exercise for lumbar segmental instability in patients with mechanical low back pain: a randomized placebo controlled crossover study. N Am J Med Sci.

[17] McAllister MJ, Hammond KG, Schilling BK, Ferreria LC, Reed JP, Weiss LW (2014). Muscle activation during various hamstring exercises. J Strength Cond Res.

[18] Shivji F, Tsegaye M (2013). Cauda equina syndrome: the importance of complete multidisciplinary team management. BMJ Case Rep.

[19] Magni G, Calderon C, Rigatti-Luchini S, Merskey H (1990). Chronic musculoskeletal pain and depressive symptoms in the general population. An analysis of the 1st National Health and Nutrition Examination Survey data. Pain.

[20] Bhagdewani N, Lakhwani M, Phansopkar P (2022). The bad contretemps causes mid-shaft tibia and fibula fracture with traumatic amputation of 2nd toe: a case report. Med Sci.

[21] Bais N, Phansopkar P, Bele A, Wadhokar O, Arora S, Chitale N (2021). A physiotherapy approach in lumbar spondylosis and PIVD in a 44 year old male patient. J Med Pharm Allied Sci.

